# Gene Expression of Pregnancy Neutrophils Differs for Protease versus Lipopolysaccharide Stimulation

**DOI:** 10.3390/ijms23094924

**Published:** 2022-04-28

**Authors:** Scott W. Walsh, Marwah Al Dulaimi, Jerome F. Strauss

**Affiliations:** Department of Obstetrics and Gynecology, Virginia Commonwealth University, Richmond, VA 23298-0034, USA; marwah.aldulaimi@vcuhealth.org (M.A.D.); jerome.strauss@pennmedicine.upenn.edu (J.F.S.III)

**Keywords:** preeclampsia, pregnancy, neutrophils, gene expression, protease-activated receptor 1, elastase, lipopolysaccharide

## Abstract

Neutrophils, which extensively infiltrate maternal systemic blood vessels in preeclampsia, express protease-activated receptor 1 (PAR-1) but only during pregnancy. Neutrophils are generally considered to be non-specific in their response, but the pregnancy-specific expression of PAR-1 could result in a gene expression profile unique to pregnancy, which could help explain why the maternal inflammatory response in preeclampsia is systemic rather than localized. We sought to determine if gene expression of pregnancy neutrophils would differ if stimulated by a protease versus bacterial lipopolysaccharide (LPS). We isolated neutrophils from normal pregnant women at 30 weeks’ gestation and cultured them with elastase or LPS. We used elastase because it is a protease elevated in women with preeclampsia, and it activates pregnancy neutrophils via PAR-1. RNA was isolated from the neutrophils for sequencing of the transcriptomes. We discovered many differences in the gene expression profiles. For example, exposure to elastase resulted in three times more uniquely expressed genes than LPS, and the number of significantly differentially upregulated and downregulated genes was greater for elastase. Analysis of canonical pathways revealed similarities for innate immunity but also differences. LPS treatment enriched more pathways, but elastase activated more genes in each pathway. Elastase treatment enriched the MAPK signaling pathway, whereas LPS did not. This is significant because MAPK is a key mediator of transcriptional responses. These findings indicate that protease stimulation of pregnancy neutrophils results in a different profile than stimulation with LPS, which may help explain why the sterile inflammatory response of preeclampsia is systemic and unique to pregnancy.

## 1. Introduction

Neutrophils, as part of the innate immune system, are important sentinels to protect the body from wound infections. As such, their response is localized and generally considered to be non-specific. However, in women with preeclampsia, there is extensive systemic infiltration of neutrophils into the mother’s blood vessels [1,2,3] which initiates a sterile inflammatory response very different from that of a wound infection. This raises the question if this different response is mediated by a difference in gene expression.

Pregnancy neutrophils are unique in that they express protease-activated receptor 1 (PAR-1), which is not expressed in neutrophils of non-pregnant subjects [4,5]. This means pregnancy neutrophils can be activated by a mechanism (proteases) that is specific to pregnancy, which could result in a neutrophil gene expression profile unique to pregnancy. There are at least three proteases elevated in preeclampsia that activate PAR-1, neutrophil elastase, matrix metalloproteinase 1 (MMP-1) and thrombin [6,7,8,9,10,11,12]. We have previously shown that elastase and MMP-1 activate nuclear factor-kappa B (NF-κB) in pregnancy neutrophils via PAR-1 to stimulate inflammatory response [6].

In this study, we sought to determine if the gene expression profile of pregnancy neutrophils differs if stimulated by a protease that activates PAR-1 versus lipopolysaccharide (LPS), a bacterial product that activates the innate immune system.

## 2. Results

**Figure 1:**
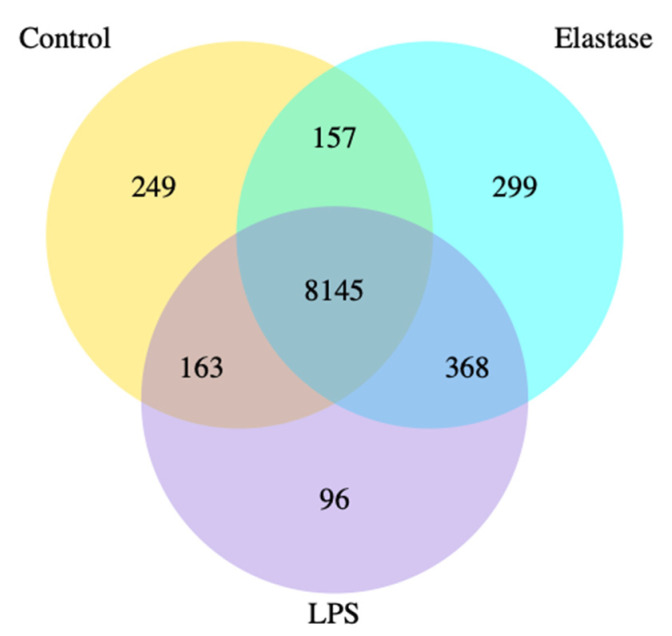
Co-expression Venn diagram. Pregnancy neutrophils were obtained at approximately 30 weeks’ gestation and treated with elastase or lipopolysaccharide (LPS). Elastase treatment resulted in 3 times more uniquely expressed genes than LPS. A total of 299 were uniquely expressed with elastase as compared to 96 for LPS.

Figure 1 shows the co-expression Venn diagram for the number of genes that are uniquely expressed for each treatment group, with the overlapping regions showing the number of genes that are co-expressed between each group for pregnancy neutrophils treated with elastase or LPS. Elastase resulted in three times more uniquely expressed genes than LPS. A total of 299 were uniquely expressed with elastase as compared to 96 for LPS.

**Figure 2:**
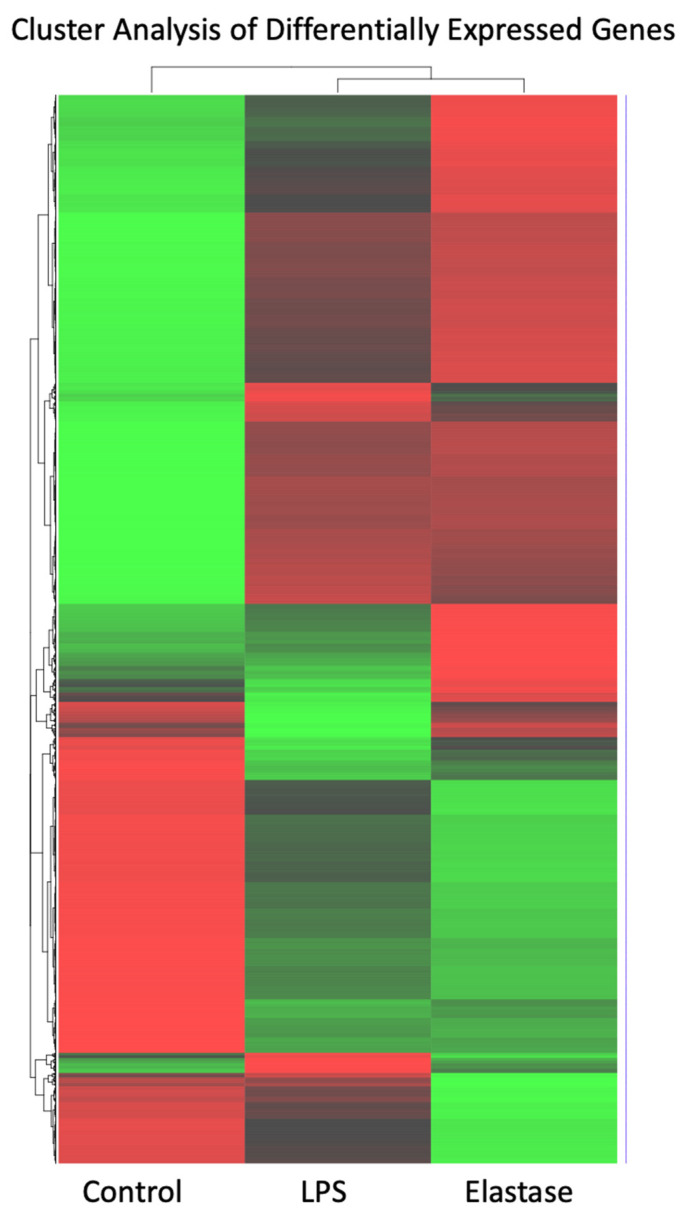
Cluster analysis heatmap of differentially expressed genes with high (red) expression levels and genes with low (green) expression levels. Cluster analysis of high and low expressed genes revealed significant differences. Elastase and LPS were clearly different from control, but they were also clearly different from each other.

Figure 2 shows a heatmap for the cluster analysis of genes with high (red) expression levels and genes with low (green) expression levels. The elastase treatment group was almost a mirror image of untreated neutrophils: Genes with low expression in the control group were highly expressed in neutrophils treated with elastase, and those with high expression in the control group showed low expression in the elastase-treated neutrophils. Pregnancy neutrophils treated with LPS were clearly different from both control neutrophils and those exposed to elastase.

**Figure 3:**
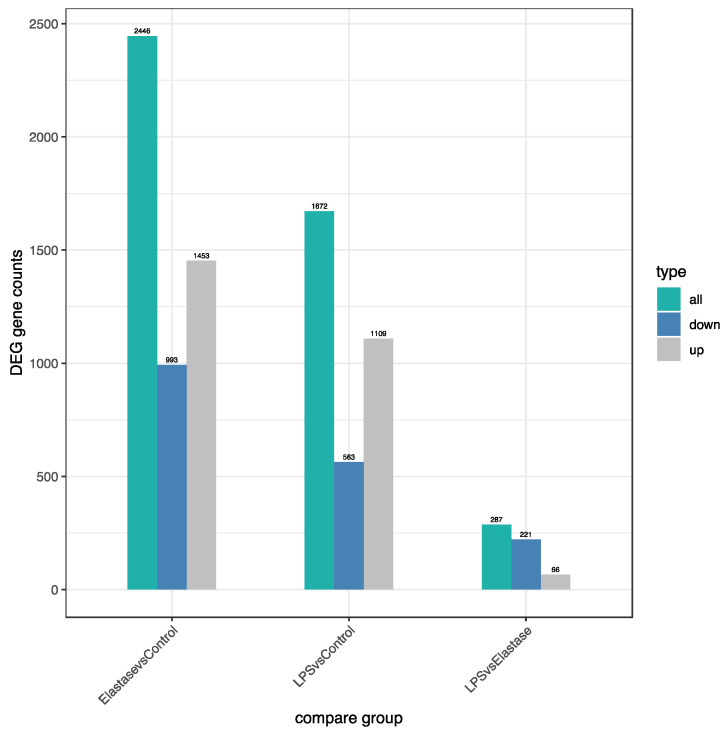
Number of significantly differentially regulated genes (DEG) either upregulated or downregulated by treatments. Elastase upregulated approximately 30% more genes than LPS and downregulated approximately 75% more genes than LPS. Overall, elastase differently regulated more genes than LPS.

**Figure 4:**
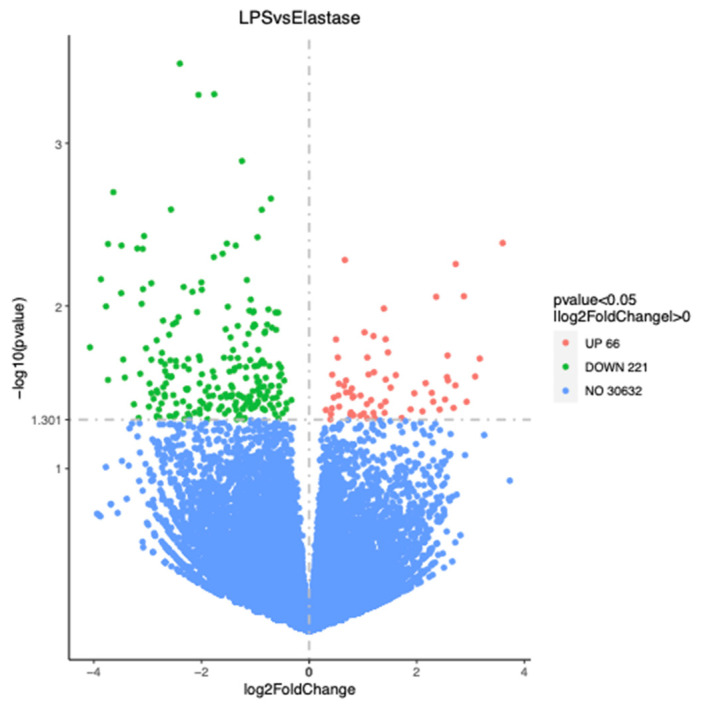
Volcano plot for significantly differentially regulated genes comparing elastase with LPS. Elastase upregulated 66 more genes than LPS and downregulated 221 more genes than LPS.

Figure 3 shows the number of significantly differentially regulated genes that were either upregulated or downregulated by treatments. Overall, treatment with elastase resulted in more upregulated and more downregulated genes than treatment with LPS. Elastase caused 344 more genes to be upregulated than LPS as compared to the control group, and 430 more to be downregulated. When elastase was compared to LPS, elastase caused 221 more genes to be downregulated and 66 more to be upregulated. Figure 4 presents the volcano plot for LPS versus elastase treatment for significantly differentially expressed genes.

The Gene Ontology Enrichment Analysis for significantly upregulated molecular function pathways is presented in Table 1. The most significantly upregulated pathways (cytokine receptor binding, cytokine activity, chemokine activity, chemokine receptor binding) were similar for elastase and LPS treatment in order of significance and number of genes involved in each pathway. However, after the first four pathways, the order of significance, number of genes, and number of pathways was different. In total, 11 pathways were upregulated by elastase treatment and 17 by LPS. There were no significantly downregulated pathways.

Table 2 shows the KEGG (Kyoto Encyclopedia of Genes and Genomes) Enrichment Analysis, which identifies significantly enriched metabolic and signal transduction pathways associated with differentially expressed genes related to biological functions. KEGG pathways related to disease information are not shown because they did not pertain to preeclampsia. The most significantly upregulated pathways, TNF signaling and IL-17 signaling, were similar for elastase and LPS treatment, although the number of genes for each pathway was greater for elastase. Thereafter, the pathways differed in order of significance, the number of genes and the number of pathways. Elastase upregulated 17 metabolic pathways and LPS upregulated 21. Of significance, elastase upregulated the MAPK signaling pathway but LPS did not. There were no significantly downregulated pathways.

## 3. Discussion

In this study, we show that the gene expression profile of pregnancy neutrophils differs depending on whether they are stimulated by a protease versus LPS. Different gene expression profiles may help explain why the pregnancy inflammatory response in preeclampsia is so different from the innate inflammatory response to localized infection. With a wound, the inflammatory response is usually restricted to the site of injury, and neutrophils infiltrate in response to bacterial or other microbial infection. In preeclampsia, the neutrophil inflammatory response is systemic, affecting the mother’s entire circulation. In a wound, neutrophils infiltrate deep into the tissue killing, trapping, and phagocytosing bacteria. In preeclampsia, neutrophils primarily adhere and flatten onto the endothelium and only infiltrate as deep as the vascular smooth muscle [1,2,3]. In a wound, neutrophil infiltration is followed by monocyte infiltration, and the subsequent differentiation of monocytes into macrophages, which assist neutrophils in cleaning up the infection site and start recruiting fibroblasts to heal the wound. In preeclampsia, vascular infiltration is limited to neutrophils; monocytes and lymphocytes do not infiltrate the mother’s blood vessels [13]. This selective neutrophil infiltration appears to be regulated by interleukin-17 (IL-17), which is increased in the mother’s circulation before clinical symptoms appear and selectively stimulates vascular expression of neutrophil chemokines as opposed to monocyte chemokines [14]. Systemic neutrophil activation and production of superoxide in preeclamptic women has been likened to sepsis [15], but the inflammation of preeclampsia is very different. Sepsis involves systemic infection, whereas preeclampsia is a sterile systemic inflammatory response.

It is evident that the neutrophil inflammatory response in preeclampsia is very different from the action of neutrophils to protect the body from infection. In this study, we found many differences in gene expression induced by elastase versus LPS. Venn diagram analysis revealed almost 300 uniquely expressed genes when pregnancy neutrophils were stimulated with elastase, whereas LPS resulted in less than 100. Heatmap cluster analysis for genes with high expression and low expression revealed very different patterns for elastase and LPS. The number of differentially regulated genes (DEG) were also different for these two activators. Elastase upregulated approximately 30% more genes than LPS and downregulated approximately 75% more than LPS. Overall, elastase differently regulated more genes than LPS.

When canonical pathways for Gene Ontology Enrichment Analysis for molecular function and KEGG Enrichment Analysis for metabolic pathways and transcription factors were analyzed, both differences and similarities were evident. The most significantly upregulated pathways were the same for both elastase and LPS. For molecular function, the first four pathways, cytokine receptor binding, cytokine activity, chemokine activity and chemokine receptor binding, were significantly enriched for both. For KEGG analysis, the first two pathways, TNF signaling and IL-17 signaling, were the same. It is not surprising that pathways related to innate immunity would be the same, since neutrophils are activated by both elastase and LPS. However, after this, the molecular function and metabolic pathway enrichments differed in terms of the order of significance, the number of genes involved and even different pathways and the number of pathways enriched.

Clearly, protease activation and bacterial LPS activation produce different gene profiles, but how this occurs is not known. Certain epigenetic mechanisms such as DNA methylation are not likely to be involved because the genomic DNA of circulating neutrophils is de-methylated [16]. Thus, differences in gene expression are more likely regulated by differences in signal transduction pathways and the regulation of transcription factor activity. This notion is supported by differences in the pathways enriched for KEGG metabolic and signal transduction. The number of pathways, the pathways enriched, and number of genes in the pathway differed for elastase and LPS. LPS enriched more pathways than elastase, but for pathways enriched by both, elastase consistently activated more genes. LPS enriched five pathways not enriched by elastase, but of note, elastase significantly enriched the MAPK signaling pathway, whereas LPS did not. This is an important difference because MAPK is a key mediator of transcriptional responses and controls gene expression in a number of ways, including phosphorylation and the regulation of transcription factors [17]. MAPK is involved in many metabolic and signal transduction pathways, so this kinase could account for differences in gene expression profiles. Our findings show that pregnancy neutrophils can express different gene profiles depending on the stimulus. The ability of neutrophils to express distinct transcriptional differences has also been shown to apply to neutrophils obtained from non-pregnant subjects with different chronic inflammatory states [18].

Circulating neutrophil elastase, MMP-1 and thrombin are all elevated in preeclampsia [6,7,8,9,10,11,12]. MMP-1 is significantly elevated 10 weeks before clinical symptoms of preeclampsia appear, a time when the women are thought to have a normal pregnancy. In contrast, neutrophil elastase and thrombin are not elevated until after the manifestation of symptoms. This suggests that MMP-1 may be responsible for the initial activation of neutrophils, but once started, neutrophil activation becomes a feed forward process accelerated by MMP-1, neutrophil elastase and thrombin. This is consistent with the progressive worsening of clinical symptoms in preeclamptic women.

Neutrophils constitutively express PAR-2 in both pregnant and non-pregnant individuals [4]. However, PAR-2 does not appear to play an important role in pregnancy based on the observation that targeted inhibition of PAR-1 alone is sufficient to prevent protease activation of NF-κB and expression of inflammatory genes in pregnancy neutrophils [6].

The control of neutrophil gene expression in preeclampsia is far more complex than shown in this study comparing elastase with LPS. In preeclampsia, multiple factors are potentially involved. Lipid peroxides, which are secreted by the placenta [19,20], are potent activators of neutrophils [21,22,23]. Lipid peroxides induce expression of cyclooxygenase (COX-2) [24] and stimulate neutrophil production of superoxide, tumor necrosis factor-alpha (TNFα) and thromboxane [22,23]. Maternal circulating levels of TNFα are increased [25,26,27] and activate neutrophils [28,29,30]. Hematopoietic cytokines, granulocyte-macrophage colony-stimulating factor (GM-CSF) and granulocyte colony-stimulating factor (G-CSF), which activate and promote the expansion of neutrophil lineages, are elevated in preeclampsia [31,32]. Chemokines, such as interleukin-8 (IL-8) produced by neutrophils and expressed in vascular smooth muscle cells under the influence of IL-17 [14], are additional activators [30,33,34,35,36]. Moreover, damage-associated molecular patterns (e.g., high-mobility group box-1 protein (HMGB1) and the extra domain A of fibronectin (EDA)) released from tissues damaged in the response to preeclampsia can also activate neutrophils [37,38,39]. Each of these factors activate neutrophils by different receptors and thus may have their own unique impact on the expression of genes, the combination of which results in the overall transcriptome profile of neutrophils in preeclampsia [16].

## 4. Materials and Methods

### 4.1. Study Subjects

Gestational age matched blood samples were collected at 30 weeks’ gestation from a multi-racial/ethnic population of women with normal pregnancy who went on to deliver at term (n = 9). The Office of Research Subjects Protection of Virginia Commonwealth University approved this study (HM20009145). All subjects gave informed consent, and the procedures followed were in accordance with institutional guidelines. Clinical characteristics of the patients are given in Table 3.

### 4.2. Neutrophil Cell Culture and RNA Sequencing

First, 2·10 mL heparin tubes of blood were collected. Lymphocytes and monocytes were separated from granulocytes (96% of which are neutrophils) by Histopaque (1077/1119) density gradient centrifugation according to the manufacturer’s protocol (Sigma Aldrich, St. Louis, MO, USA) and as previously described [2,23,40]. Neutrophils were seeded at an average of 5,000,000 cells per mL in Falcon 4-well cell culture slides (#354104) and cultured in Iscove’s Modified Dulbecco’s Medium supplemented with 10% fetal bovine serum and 1% antibiotics and antimycotics (100 U/mL penicillin, 100 µg/mL streptomycin, 0.25 µg/mL amphotericin B) at 37 °C in a humidified 5% CO_2_ atmosphere. Neutrophils were incubated with the following treatments for 2 h: (1) control media; (2) elastase (0.33 U/mL, Sigma Aldrich); (3) LPS (200 ng/mL, Sigma Aldrich). We used elastase because it is a neutrophil product that is elevated in women with preeclampsia, and we previously showed that it activates NF-κB in pregnancy neutrophils via PAR-1 [6]. We used LPS because it is a bacterial product that activates neutrophils through a different mechanism, that is, via toll-like receptors. RNA was isolated from neutrophils using TRIzol Reagent (ThermoFisher Scientific, Wilmington, DE) according to the manufacturer’s protocol. RNA concentrations were measured, and their quality assessed using a NanoDrop 2000 spectrophotometer (ThermoFisher Scientific). Total RNA (0.5 µg/20 µL) was sent to Novogene Corporation, Inc. (Sacramento, CA) for Human mRNA Sequencing. RNA sample quality was determined by Novogene before proceeding with mRNA library preparation (poly A enrichment). A paired-end 150 bp sequencing strategy was used to sequence the samples using Illumina NovaSeq 6000 Sequencing Platform. The resulting data were checked for quality before bioinformatic analyses. The hg38 genome was used as the reference genome for gene alignment. Novogene provided the bioinformatics analysis for the RNA-seq Quantification Analysis Report along with publication-ready results.

## 5. Conclusions

In this study, we demonstrated that the gene expression profile of pregnancy neutrophils differs when stimulated by a protease elevated in preeclampsia versus stimulated by LPS, a bacterial product that activates the innate immune system. The pregnancy-specific expression of PAR-1, which allows neutrophils to respond to activators (proteases) that they otherwise would not recognize, may explain why the inflammatory response in preeclampsia is systemic and has unique features.

## Figures and Tables

**Table 1:** Gene Ontogeny Molecular Function Enrichment Pathways.

Elastase vs. Control		
***Pathway Description***	***Number of Genes***	***padj***
cytokine receptor binding	40	4.44 × 10^−8^
cytokine activity	34	4.44 × 10^−8^
chemokine activity	12	0.00023355
chemokine receptor binding	13	0.0004327
MAP kinase phosphatase activity	7	0.00044775
receptor regulator activity	42	0.00200428
receptor ligand activity	40	0.00200428
MAP kinase tyrosine/serine/threonine phosphatase activity	6	0.00266583
CCR chemokine receptor binding	8	0.00933415
growth factor receptor binding	17	0.01480743
growth factor activity	17	0.04707246
**LPS vs. Control**		
***Pathway Description***	***Number of Genes***	***padj***
cytokine receptor binding	39	5.63 × 10^−10^
cytokine activity	31	2.43 × 10^−8^
chemokine activity	12	1.62 × 10^−5^
chemokine receptor binding	13	3.10 × 10^−5^
CCR chemokine receptor binding	9	0.00031289
receptor ligand activity	37	0.00031289
receptor regulator activity	37	0.00131844
MAP kinase tyrosine/serine/threonine phosphatase activity	5	0.01543015
14-3-3 protein binding	7	0.01543015
G-protein coupled receptor binding	23	0.01698056
MAP kinase phosphatase activity	5	0.01759535
RNA polymerase II proximal promoter sequence-specific DNA binding	33	0.02366222
growth factor receptor binding	14	0.03717801
proximal promoter sequence-specific DNA binding	33	0.03773685
RNA polymerase II distal enhancer sequence-specific DNA binding	10	0.04702544
lipoprotein particle binding	6	0.04702544
protein–lipid complex binding	6	0.04702544

The adjusted *p*-value (padj), which is the transformation of the *p*-value after accounting for multiple testing, was used to determine statistical significance for these pathways. The padj values were calculated by Novogene.

**Table 2:** KEGG Metabolic Enrichment Pathways.

Elastase vs. Control		
***Pathway Description***	***Number of Genes***	***padj***
TNF signaling pathway	34	1.32 × 10^−13^
IL-17 signaling pathway	27	1.03 × 10^−10^
NOD-like receptor signaling pathway	37	1.03 × 10^−10^
C-type lectin receptor signaling pathway	24	6.11 × 10^−7^
NF-kappa B signaling pathway	21	4.46 × 10^−6^
Cytokine-cytokine receptor interaction	35	8.26 × 10^−5^
Toll-like receptor signaling pathway	17	0.000551
RIG-I-like receptor signaling pathway	13	0.00103201
Chemokine signaling pathway	25	0.00135461
Cytosolic DNA-sensing pathway	11	0.00221418
AGE-RAGE signaling pathway in diabetic complications	16	0.00281374
Necroptosis	21	0.00440828
Neurotrophin signaling pathway	17	0.0127872
JAK-STAT signaling pathway	19	0.01428866
Th17 cell differentiation	14	0.01622074
MAPK signaling pathway	30	0.02969086
Th1 and Th2 cell differentiation	12	0.03381456
**LPS vs. Control**		
***Pathway Description***	***Number of Genes***	***padj***
TNF signaling pathway	27	1.14 × 10^−9^
IL-17 signaling pathway	23	1.01 × 10^−8^
NF-kappa B signaling pathway	22	9.69 × 10^−8^
C-type lectin receptor signaling pathway	22	6.87 × 10^−7^
NOD-like receptor signaling pathway	27	1.98 × 10^−6^
Cytokine-cytokine receptor interaction	31	6.38 × 10^−5^
RIG-I-like receptor signaling pathway	13	0.00018154
Toll-like receptor signaling pathway	16	0.0002092
Chemokine signaling pathway	23	0.00065273
AGE-RAGE signaling pathway in diabetic complications	14	0.00820716
Cytosolic DNA-sensing pathway	9	0.00845507
JAK-STAT signaling pathway	17	0.01259687
Th17 cell differentiation	13	0.01280896
B cell receptor signaling pathway	11	0.01290435
Necroptosis	17	0.01834355
Adipocytokine signaling pathway	10	0.0190038
T cell receptor signaling pathway	13	0.0190038
Neurotrophin signaling pathway	14	0.02891941
Th1 and Th2 cell differentiation	11	0.03029502
Ferroptosis	7	0.03301585
Apoptosis	15	0.03522501

The adjusted *p*-value (padj), which is the transformation of the *p*-value after accounting for multiple testing, was used to determine statistical significance for these pathways. The padj values were calculated by Novogene.

**Table 3:** Clinical Characteristics of Patients.

Variable	Normal Pregnant n = 9
Maternal age (years)	29.8 ± 4.4
Pre-pregnancy BMI (kg/m^2^)	29.5 ± 11.3
BMI at sample collection (kg/m^2^)	34.5 ± 11.4
Systolic blood pressure at 30 Weeks (mmHg)	109 ± 13
Diastolic blood pressure at 30 Weeks (mmHg)	68 ± 11
Primiparous	1
Multiparous	8
Race	
White	4
Black	3
Hispanic	1
Asian	1
Type of Delivery	
C-section	2
Vaginal	7
Gestational age at sample collection (weeks)	29.6 ± 2.6
Gestational age at delivery (weeks)	38.7 ± 2.4
Infant birth weight (grams)	3288 ± 322

Values are mean ± SD.

## Data Availability

Complete tabular data presented in this study for gene ontogeny pathways and gene expression are available on request from the corresponding author.
